# Prognostic Assessment of Interim F18-Fluorodeoxyglucose Positron Emission Tomography in Esophageal Cancer Treated With Chemoradiation With or Without Surgery

**DOI:** 10.7759/cureus.29086

**Published:** 2022-09-12

**Authors:** Sophie Lavertu, Maroie Barkati, Sylvain Beaulieu, Jocelyne Martin, Marie-Pierre Campeau, David Donath, David Roberge

**Affiliations:** 1 Radiation Oncology, Hôtel-Dieu de Lévis, Centre Intégré de Santé et de Services Sociaux de Chaudière-Appalaches (CISSS-CA), Lévis, CAN; 2 Radiation Oncology, Centre Hospitalier de l'Université de Montréal (CHUM), Montréal, CAN; 3 Nuclear Medicine, Hôpital Maisonneuve-Rosemont, Centre Intégré Universitaire de Santé et de Services Sociaux (CIUSSS) de l'Est-de-l'Ile-de-Montréal, Montréal, CAN; 4 Thoracic Surgery, Centre Hospitalier de l'Université de Montréal (CHUM), Montréal, CAN

**Keywords:** prognosis, metabolic response, 18fdg-pet, concurrent chemoradiation therapy, locally advanced esophageal cancer

## Abstract

Purpose

This study aimed to evaluate if the F18-fluorodeoxyglucose positron emission tomography (F18-FDG PET) response after two weeks of chemoradiation for locoregionally advanced esophageal cancer (staged Tumor (T) 3 and/or Nodes (N)+ Metastases (M) 0) was linked to the pathologic response for patients undergoing surgery, to disease-free survival (DFS) or overall survival (OS).

Materials and Methods

Between March 2006 and September 2017, 40 patients were prospectively enrolled in our study, gave written consent, and had PET scans performed before treatment and after two weeks of chemoradiation. One patient did not undergo his two-week PET without informing study coordinators and was excluded from analyses.

Results

The median age at diagnosis was 62 years. Seventy-two percent of patients had N+ disease. Median OS for the entire group was 24 months. Five-year overall survival was 17%. Survival curves for patients with no PET response, minor PET response, or good PET response overlapped and were not statistically different. For the 25 patients who underwent surgery, the positive predictive value (PPV) of the PET response relative to the pathologic response was 75% and the negative predictive value (NPV) was 62%. In study patients, the crude recurrence rate was 68% and there was no correlation between PET response and DFS.

Conclusion

In our study, interim PET response after two weeks of chemoradiation for locoregionally advanced esophageal cancer was not predictive of outcome or pathologic response. Based on our data and current literature, interim PET should not be used to alter treatment (whether to escalate neo-adjuvant treatment or omit surgery).

## Introduction

Esophageal cancer is a major health problem and the prognosis is guarded. Curative treatment for locoregionally advanced disease, namely chemoradiation, followed or not by surgery, is toxic. In the last 25 years, F18-fluorodeoxyglucose positron emission tomography (F18-FDG PET) became a standard component of the workup of these cancers to establish the stage of the disease and is very useful in detecting distant metastases not found by standard tomodensitometry [1]. Earlier studies have reported that response to FDG-PET three to five weeks after the end of chemoradiotherapy correlated with pathological response, disease-free survival (DFS), and overall survival (OS) [2-5].

At the time we designed the study, experts in the field had hypothesized that FDG-PET response after two weeks of chemoradiation in locoregionally advanced esophageal cancer (Tumor (T) 3 and/or Nodes (N) +) without distant metastases (M0) would be predictive of outcome [2,5-7]. If this were the case, early FDG-PET response could eventually be used to modify treatment plans for future patients, for instance, stopping chemoradiation or omitting surgery in patients with suboptimal positron emission tomography (PET) response, in the purpose of minimizing toxicity in such patients, stopping chemoradiation and directly moving to surgery to reduce toxicity but still hoping for some benefit from surgery, or escalating chemoradiation in good PET responders, expecting an even better response with more intense chemoradiation (in patients undergoing surgery or not). 

In our study, we wanted to include a broad population, hence recruiting patients undergoing surgery or treated with chemoradiotherapy alone. We planned to compare FDG-PET response with the pathological response for surgical patients, and PET response to recurrence rate, DFS, and OS in all patients.

This article was previously presented in abstract forms and as a poster presentation at the American Society for Therapeutic Radiation and Oncology (ASTRO) 61st Annual Meeting on September 15, 2019, in Chicago, and as an oral presentation at the Canadian Association of Radiation Oncology (CARO) Annual Meeting on October third, 2019 in Halifax, Nova Scotia, Canada.

## Materials and methods

Before prospectively enrolling patients (40 planned) between March 15, 2006, and September 20, 2017, the local Institutional Review Board (IRB), Montreal University Hospital Center (CHUM) Scientific Evaluation and Ethical Research Committees had issued approval number ND (Notre-Dame)-05-084 to the study and consent form. Interim PET exams were performed off-site in another hospital facility (although part of the same institution).

Pretreatment evaluations included a complete history and physical examination, biopsy, chest and abdominal (+/- neck) tomodensitometry, endoscopic ultrasound (except for tumors with very severe stenosis), PET, bronchoscopy if indicated, blood work (complete blood count, renal and liver function tests) and Eastern Cooperative Oncology Group (ECOG) status.

Female (non pregnant) and male patients 18 years and older, with an ECOG status 0 or 1, with an esophageal cancer, staged T3-4, N0-1, M0-1a or T1-2, N1, or M1a according to the 2002 sixth Tumor Nodes Metastases (TNM) American Joint Committee for Cancer (AJCC) esophageal staging classification, without synchronous cancer (except for basal or squamous cell carcinoma of the skin), without contraindication to chemoradiation, and capable of informed consent, were included. Patients with distant metastases (M1b) or treated with surgery only (T1-T2, N0, M0) were excluded.

The chemotherapy agents given were at the discretion of the medical oncologist in charge of the patient and depended on the patient’s general health condition as well as the standard(s) in use at the time of treatment. Radiotherapy plans were either three-dimensional (3D) conformal fields or, as the technique used in our department evolved, intensity-modulated radiation therapy (IMRT), with megavoltage linear accelerators. The doses prescribed were between 41.4 grays (Gy)/23 fractions and 50 Gy/25 fractions.

Patients analyzed had their interim PET scan exam performed somewhere between two to three weeks of chemoradiotherapy. FDG-PET exams were done after at least six hours of fasting, with a blood glucose level under 10 mmol/liter (using insulin if needed), one hour and a half after an intravenous dose of F18-fluorodeoxyglucose adjusted to the patient’s weight (average dose of 410 megabecquerels). The interpretation was performed by experienced physicians, and included the standardized uptake value (SUV) of the primary tumor, the size of the primary tumor (for most patients), the nodal extent of the disease, and a search for distant metastases. PET responses were classified as a good response, minor response, or no response to assess the link between these responses and outcome measures. Good response was characterized by a decrease of more than one-half in SUV, mostly with size reduction of the primary tumor (but a few reports missed size specifications), some with a decrease in SUV and/or size of involved nodes as well. Patients with minor response included those with SUV reduction of less than one-half (though one reported missing SUV value), mostly with a decrease of less than one-half in the esophageal tumor size (but still missing a few size descriptions), and again a few with some reduction of nodal SUV/and or size. No response was defined as no change or an increase in SUV, with primary tumor size the same or larger in those with size measurements, and a few with new nodal involvement as well. 

Follow-up was calculated from the first day of chemoradiation. The Kaplan-Meier method was used to assess OS and DFS and survival curves compared with the log-rank test employing Prism software (GraphPad, San Diego, California, now part of Dotmatics, Boston, Massachusetts). The positive predictive value (PPV) and negative predictive value (NPV) were calculated to evaluate the relationship between PET response and pathologic response in patients undergoing surgery.

## Results

Thirty-nine patients were included in the final data analysis. An additional 11 patients consented to study participation (having signed informed consent as well) but later declined the interim PET exam (one without informing study coordinators), typically because of treatment-related fatigue and/or toxicity. The median age at diagnosis was 62 (range: 31-79). Eighty-seven percent (%) of patients were male. Seventy-two percent of patients had adenocarcinomas, and 74 % had disease in the distal esophagus and/or cardia. Thirty-nine percent had moderately differentiated tumors. Seventy-two percent of patients had N+ disease (some staged M1a as per the 2002 TNM staging).

Forty-one percent of patients had 5-Fluorouracil/Cisplatinum (5FU-DDP) chemotherapy (most prior to 2011), otherwise, patients were treated with Carboplatin/Paclitaxel (Carbo-Taxol) chemotherapy, typically with 41.4 Gy/23 fractions in the neoadjuvant setting [8], and with 50 Gy/25 fractions in the radical setting. Twenty-five patients (64%) underwent surgery. Patients, tumor, and treatment features are shown in Table 1.

**Table 1:** Patient, tumor and treatment features. N = nodes; M = metastasis; T = tumor; 5FU = 5-fluorouracil; DDP = Cisplatin; Carbo =Carboplatin; Taxol=Paclitaxel.

Characteristics		Overall (n=39)
Median age (range)		62 (31-79)
Sex (%)	Female	5 (12.8)
	Male	34 (87.2)
Histology (%)	Adenocarcinoma	28 (71.8)
	Squamous cell	11 (28.2)
Location in the esophagus (%)	Cervical and/or upper	7 (17.9)
	Middle	3 (7.7)
	Lower and/or cardia	26 (66.7)
	Mixed middle/lower	3 (7.7)
Grade (cell differentiation) (%)	Good	3 (7.7)
	Moderate	15 (38.5)
	Poor	6 (15.4)
	Mixed	12 (30.8)
	Undefined	3 (7.7)
N stage	N0	11 (28.2)
	N+ (including M1a as per 2002 TNM)	28 (71.8)
Chemotherapy	5FU-DDP	16 (41)
	Carbo-Taxol	23 (59)
Surgery	Yes	25 (64.1)
	No	14 (35.9)

No patient had developed distant metastases on interim PET. As of January 25 2019, we had survival data for 38 patients and recurrence data for 37. Median OS for the entire group was 24 months. Five-year OS was 17%. The crude recurrence rate was 68%. We could not establish any relationship between age or sex, and PET response or outcomes. Neither PET response nor clinical outcomes were associated with histological type, tumor location, N stage or type of chemotherapy.

**Figure 1:**
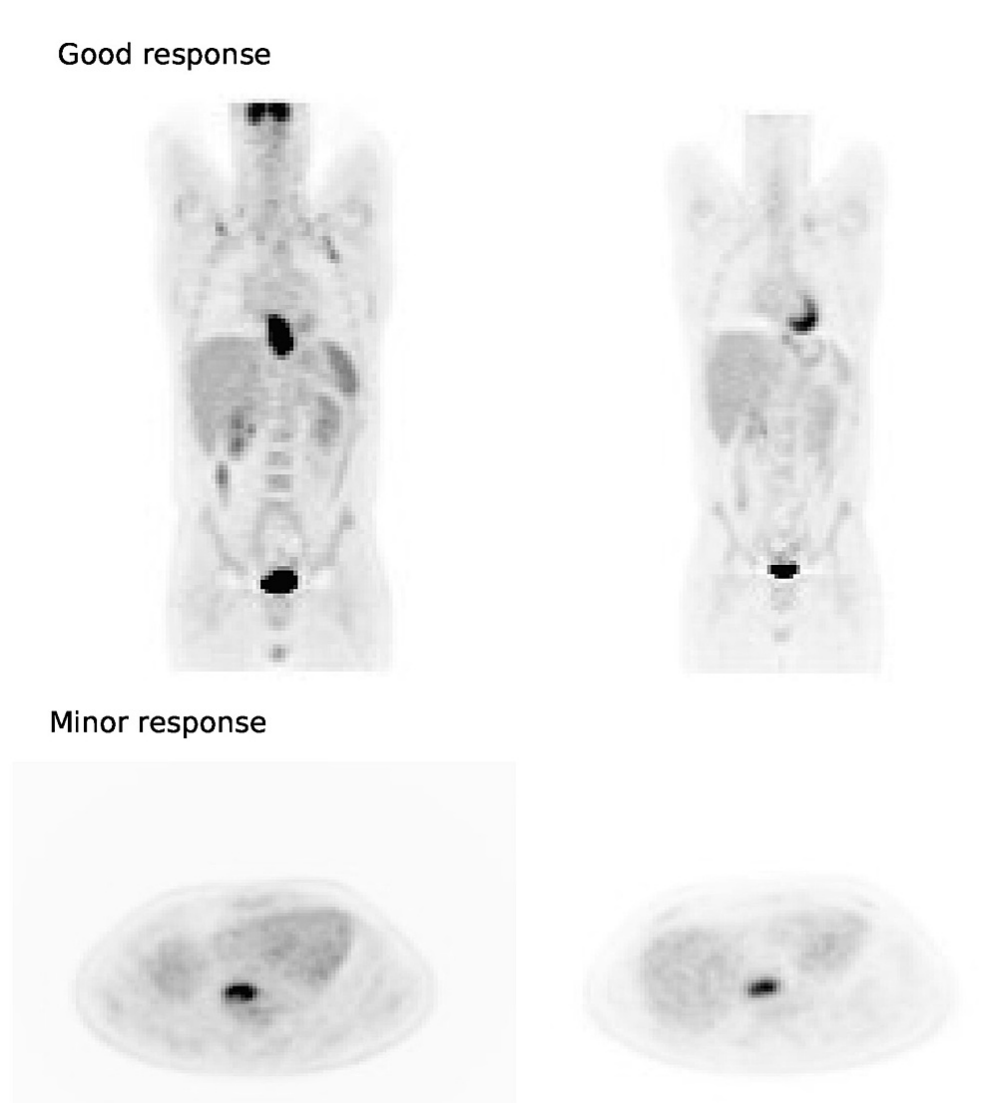
Images of good and minor responses on positron emission tomography after two weeks of chemoradiation.

In the 25 patients who underwent surgery, 14 (56%) showed good pathological response to chemoradiation, and among them, eight showed a complete response (32% of the entire surgical group). The PPV of the PET response relative to the pathologic response was 75%, and the NPV was 62%. The PPV of the PET response relative to complete pathologic response was 42%. Examples of good and minor metabolic responses can be seen in Figure 1.

**Figure 2:**
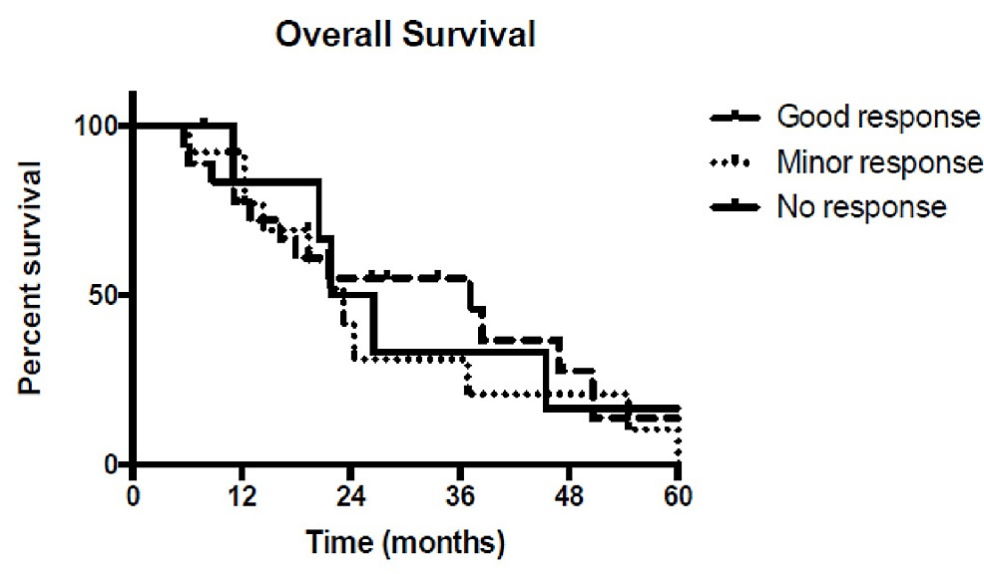
Survival curves according to positron emission tomography response after two weeks of chemoradiation.

Survival curves for patients with no PET response, minor PET response or good PET response overlapped (as shown in Figure 2) and were not statistically different . Finally, there was no correlation between PET response and DFS.

## Discussion

FDG-PET is part of the initial work-up of esophageal cancer as in many other tumor types, and its use to detect distant metastases not shown by standard tomodensitometry is well established [1]. In general, the SUV response on PET a few weeks after the end of chemoradiation shows a good correlation with pathologic response and prognosis, as demonstrated by studies published before the start of our own study [2-5], as well as studies published afterwards [9-11]. Indeed, Flamen, et al. found statistically different median survival times in major PET responders (16.3 months) and in PET non-major responders (6.4 months) four to five weeks after completion of chemoradiation [2].

When PET is done weeks after completion of chemoradiation, it is too late to consider changes in the radiotherapy regimen. This prompted us, as suggested by experts, to design a study to evaluate the prognostic value of FDG-PET performed early in the course of chemoradiotherapy [2,5-7]. Had we found a strong correlation, we could have considered customization of treatment according to early PET response. Since our study was initiated, several reports (including reviews and one meta-analysis) have come to conclusions similar to ours [9-18]. Of note, Bollschweiler, et al. concluded in their review that PET response early in the course of radiochemotherapy has a rather good sensitivity for prediction of pathologic response whereas a too low specificity to be used in clinical practice [17]. A systematic review performed by Cremonesi, et al. has reported conflicting results [19]. Other studies have suggested that the SUV change on interim PET was predictive of pathologic response and/or locoregional recurrence, distant metastases and overall survival [20-22].

More studies of neoadjuvant chemotherapy only or induction chemotherapy before neoadjuvant chemoradiation show some link between PET response to chemotherapy a few weeks after the end of this chemotherapy (either before surgery if no chemoradiation is given or before neoadjuvant chemoradiation) and pathologic response and/or prognosis [23-27]. The noteworthy MUNICON phase II trial, in which 110 patients were evaluated, and metabolic non responders after two weeks of chemotherapy discontinued treatment and proceeded to surgery while metabolic responders continued chemotherapy before surgery, demonstrated a strong link between PET response and pathologic response, as well as a statistical significant difference in median event-free survival (29.7 months versus 14.1 months) in metabolic responders and non responders respectively [23]. Conversely, a few studies do not show such a strong enough correlation to guide treatment decisions in clinical practice in that setting [28-29].

Nowadays, there is some evidence that diffusion-weighted magnetic resonance imaging (DW-MRI) done early during neoadjuvant chemoradiotherapy may be more useful to predict pathologic complete response as compared to FDG-PET [30].

A significant limitation of this prospective study is the much longer accrual time (over 11 years) than expected (approximately two years) when writing the protocol, based on the number of patients with locoregionally advanced esophageal cancers treated in our center and taking into account a number of potential refusals. The study group was aware of the hindrance of going to another facility (Montreal Hôtel-Dieu) a few miles away from the treatment site (Notre-Dame Hospital) to get their interim PET done for patients in a limited health condition and receiving difficult treatments, though not to that extent, and it was not possible to overcome that problem. More so, 11 patients did not undergo this PET exam although having agreed to the study, because of fatigue and/or treatment toxicity. Likewise, the number of patients included in our study might seem rather limited. Some might believe we should have only included patients undergoing surgery, or patients not undergoing surgery, but the aim of the study was in fact to recruit a broad population reflecting real patients encountered in hospital clinics. The same idea pertains to evolving chemotherapy over the years, mainly for patients treated preoperatively. And excluding one or the other category of patients would have meant an even longer accrual phase as well. Unfortunately, at final analysis, we missed survival data for one patient and recurrence data for two patients, although we had thoroughly looked for such data into the ethical and legal frameworks in force in the Quebec province.

Along with this long accrual period emerged the problems of some technical differences in performing PET exams over the years and interpretation by various nuclear medicine specialists as well. Technical and measurements/interpretation issues are extensively addressed in reviews by Bollschweiler et al. [17], and Wilson et al. [18]. While writing the protocol, the intent was to use SUV threshold values, SUV delta values and possibly receiver operating characteristic curves to assess PET ability to predict outcomes. As mentioned earlier, due to technical and measurements/interpretation differences over the years, this was impossible. Nonetheless, with the data available on comparisons of the SUV of the primary tumor, the size of the primary tumor and the nodal extent of the disease on initial and interim PET scans, and the potential occurrence of distant metastases, we were able to classify PET responses as good, minor, or no response in order to assess the link between these responses and outcome measures, then reach conclusions.

## Conclusions

This study was aimed at prospectively enrolling, over a few years, about 40 patients with locoregionally advanced esophageal cancer receiving chemoradiation with or without surgery thereafter, and performing a PET exam after two weeks of chemoradiation for each patient in order to evaluate the link between the treatment response on this second PET (as compared to the initial diagnostic PET) and pathological response in patients undergoing surgery, as well as DFS and OS in all patients. Unfortunately experiencing recruitment problems (as described in the Discussion section), resulting in a long accrual time, we have finalized this study which is, in our view, practice-oriented, hence relevant.

In our study, interim PET response after two weeks of chemoradiotherapy for locoregionally advanced esophageal cancer was not predictive of pathological response nor clinical outcome, similarly as in many other publications. Based on our data, interim PET exam should not be used to alter treatment. SUV response after the end of chemoradiation appears to have a stronger correlation with prognosis than interim PET response.
